# Cross-cultural adaptation of the Voice-related Experiences of Nonbinary Individuals - VENI to Brazilian Portuguese

**DOI:** 10.1590/2317-1782/20242023170en

**Published:** 2024-05-27

**Authors:** Isabela dos Santos, Mara Behlau, Grace Shefcik, Pei-tzu Tsai, Vanessa Veis Ribeiro

**Affiliations:** 1 Curso de Especialização em Voz – CECEV, Centro de Estudos da Voz – CEV - São Paulo (SP), Brasil.; 2 Programa Associado de Pós-graduação em Fonoaudiologia – PPGFON, Universidade Federal da Paraíba – UFPB, Universidade Estadual de Ciências da Saúde de Alagoas, Universidade Estadual de Ciências da Saúde de Alagoas – UNCISAL - João Pessoa (PB), Brasil.; Universidade Federal do Rio Grande do Norte – UFRN,; 3 Department of Communicative Disorders and Sciences, San José State University - San Jose (CA), USA.; 4 Curso de Fonoaudiologia, Faculdade de Ceilândia, Universidade de Brasília – UNB - Brasília (DF), Brasil.

**Keywords:** Voice, Clinical Protocols, Self-Testing, Quality of Life, Gender Identity, Speech, Language and Hearing Sciences

## Abstract

**Purpose:**

This study aimed to translate and cross-culturally adapt the “Voice-related Experiences of Nonbinary Individuals” (VENI) to Brazilian Portuguese (BP).

**Methods:**

Cross-cultural adaptation was performed based on the combined guidelines of the World Health Organization's (WHO) Translation Recommendations and the COnsensus-based Standards for the selection of health Measurement INstruments (COSMIN). The process included five stages: a) Translation of the instrument into BP by a translator specialized in the construct and a non-specialist, both native BP speakers and fluent in English; b) Synthesis of the two translations by consensus; c) Back-translation by a translator specialized in the construct and a non-specialist, both native English speakers and fluent in BP; d) Analysis by a committee of five speech-language pathologists voice specialist and the creation of the final version; e) Pre-testing with 21 individuals from the target population, conducted virtually.

**Results:**

During the translation stage, there were disagreements regarding the title, instructions, response key, and 15 items. In the back-translation stage, there were discrepancies in the format of 12 items and the content of four items. The expert committee's analysis led to changes in the title, instructions, one option in the response key, and eight items to meet the equivalence criteria. In the pre-test, a significantly higher proportion of usual responses to the instrument was observed when compared to the non-applicable option; this is frequently observed in instrument adaptations.

**Conclusion:**

The cross-cultural adaptation of VENI into Brazilian Portuguese was successful, resulting in the “Experiências relacionadas à Voz de Pessoas Não Binárias - VENI-Br” version.

## INTRODUCTION

Vocal quality reflects the acoustic properties of sound as it travels through the vocal tract and also includes neuromuscular aspects of vocal production and control, auditory monitoring, and factors such as personality traits, personal preferences, and cultural elements^(1)^. The voice plays a crucial role in an individual's unique identity, such as in socialization, self-perception, communicative experiences, and cultural contexts. Therefore, it is essential to understand each individual's vocal needs and preferences^(1,2)^.

In the transgender population, the voice is a powerful reflection of gender identity, significantly influencing social inclusion, survival and quality of life^(3)^. Gender identity refers to the personal sense of gender as experienced by each person. This experience is unique and reflects the individual's self-awareness of aligning with or diverging from specific gender norms. Such identity may be masculine, feminine, nonbinary, or expansive^(4,5)^.

Sex and gender are often confused or used as synonyms but represent distinct concepts. Sex refers to biological attributes such as chromosomes, hormones, and reproductive organs and is typically seen as binary (male or female) and unchangeable. The female sex is associated with an XX chromosomal configuration and the male with an XY. On the other hand, gender incorporates socially and culturally constructed traits and identities, with self-identification playing a central role. This broader perspective allows for categorizations beyond the traditional binary conception of sex and gender^(4)^.

Some individuals align with the binary gender assigned at birth based on their genitalia and are called cisgender men or women in binary terminology. Conversely, individuals who do not identify with the gender assigned at birth are termed as transgender male or female. However, this binary classification is not comprehensive. Many find this classification limiting and instead identify with nonbinary gender, fluidly aligning with one, both, or neither of the binary genders. The increasing demand for speech-language pathology services, driven by the unique needs and experiences of nonbinary individuals and the broader transgender community, reflects the crucial role of voice and communication in expressing gender identity. There is a particular emphasis on gender affirmation through communication and language training^(4-6)^.

Special attention is given to voice, exploring adjustments such as gender-neutral vocal quality, fluctuations in fundamental frequency, changes in resonance, articulation, and elements of nonverbal communication^(7)^.

Nonbinary individuals have unique communicative and vocal needs, differing from those of binary individuals. This necessitates focused studies for a better understanding and more effective practices within speech-language pathology. Nonbinary individuals may seek feminine, masculine, or gender-neutral voices^(6,8)^.

The self-perception of vocal characteristics is subjective and comparative, involving emotional, personality, and experiential factors. Vocal self-assessment holds significant clinical value as it captures the client's voice perception, guiding for vocal interventions or training^(1,2)^.

To achieve a comprehensive understanding of the communicative experiences and vocal self-assessment of nonbinary individuals, specialized assessment tools are necessary^(9,10)^. The only validated instrument developed explicitly for this population is the Voice-related Experiences of Nonbinary Individuals (VENI). The VENI quantitatively assesses voice-related experiences and assists in customizing gender-affirming communication services. It has 17 items designed to strategize, monitor progress, and document changes in vocal self-perception^(6)^. Given its developed and validation in English, there is a necessity for cross-cultural adaptation and validation to enable its use in other languages and cultures. Cross-cultural adaptation is the initial step in the instrument validation process. It ensures that the original items' content and intent correspond with the linguistic and cultural context of a different language^(9-12)^. Adapting the VENI to Brazilian Portuguese will improve the understanding of the concerns, experiences, perceptions, and vocal goals of nonbinary individuals in Brazil who seek voice intervention. Also, it will facilitate the planning of vocal interventions, thus, improving outcomes for this population^(6,8)^.

Therefore, this study aims to perform the cross-cultural adaptation of the VENI to Brazilian Portuguese.

## METHODS

This is a cross-sectional study. The present research was approved by the Research Ethics Committee of the “Faculdade de Ceilândia da Universidade de Brasília – UNB” (approval number 5.845.012). The research followed all ethical guidelines outlined by Resolution No. 466/2012 and other relevant legal regulations in Brazil. All participants agreed to participate and informed consent.

The Voice-related Experiences of Nonbinary Individuals (VENI), originally developed in English, has 17 items divided into three factors: seven items related to physical issues (items 1 to 7), eight items related to functional issues (items 8, 9, 10, 11, 12, 14, 15, and 16), and two items related to emotional issues (items 13 and 17). Each item provides a response key with four options: 1 = never or rarely; 2 = sometimes; 3 = often, and 4 = usually or always. Score items within their respective factors are calculated by simple summation^(6)^.

The cross-cultural adaptation process of the VENI to Brazilian Portuguese followed the recommendations and guidelines outlined by the World Health Organization (WHO) for translation^(9)^, and the COnsensus-based Standards for the selection of health Measurement INstruments (COSMIN)^(10)^.

Following COSMIN guidelines^(10)^, a panel of expert judges was formed for the translation, back-translation, and expert review stages; also participants for the pre-test were selected. All stages were conducted virtually.

Two translators, one speech-language pathologist (SLP), and one non-specialist, both native speakers of Brazilian Portuguese (BP) and fluent in English, independently translated the instrument. They prioritized conceptual over literal translation, translating the title, instructions, response scale, and items.

Subsequently, the authors synthesized a consensus version from the two translations, resolving disagreements among the judges.

The Brazilian Portuguese version was then back-translated into English by two judges, an SLP, and a non-specialist, both native English speakers fluent in Brazilian Portuguese. A committee of five SLPs, including the translators, authors, and additional specialists, compared the back-translation to the original instrument and made consensus-based decisions on any necessary changes to ensure:

- Semantic equivalence: ensuring that words in the final version maintain the same meaning as in the original.
- Conceptual equivalence: identifying and replacing words or expressions with differing conceptual meanings across cultures.
- Idiomatic equivalence: reformulating colloquial idioms that are difficult to translate to preserve their equivalence in the target language.
- Experiential equivalence: replacing any original item with a similar item in the target culture as necessary.
- Cultural equivalence: making orthographic or grammatical adjustments to the items.
- Operational equivalence: modifying procedures related to the application of the instrument if required.

Every step was documented in a master table, which was updated upon the completion of each stage.

The final version of the instrument underwent a pre-test, where the cross-culturally adapted version was applied to the target population. An option for non-applicable responses (N/A) was added to the instrument's response key. Participants were instructed to select this option if they did not understand the item or if it did not apply to their culture. Items flagged with issues, particularly those with a high number of N/A responses, underwent review.

Eligibility criteria for the pre-test included self-identification as nonbinary and an age range between 18 and 65 years. Individuals self-reporting cognitive impairments affecting their comprehension of the instrument were excluded.

Sample size calculation used the statistical test for a confidence interval for one sample mean. Parameters included a type I error (α) of 5%, type II error (β) of 10%, and test power (K) of 90%. The estimated proportion of nonbinary individuals in Brazil, 1.19%, was used for the calculation^(13)^. Consequently, a sample size of 19 participants was required, determined using Statistica software version 11.0. Therefore, 21 nonbinary individuals, with an average age of 30.71 years (SD: 8.66) were selected for the pre-test.

Participants were recruited virtually via social and digital media channels, where an information flyer with a link to the research was distributed. When clicking, participants were directed to the Google Forms platform. Those meeting eligibility criteria and agreeing to the informed consent form would then have access to the data collection instruments, which included a participant identification questionnaire and the VENI.

Pre-test data were analyzed using SPSS software version 25.0. The Chi-Square Test was used. Additionally, the Binomial Test was utilized to compare the proportion of non-applicable responses with the proportion of habitual responses on the instrument response key (1, 2, 3, or 4) for each item.

## RESULTS

During the initial translation stage, discrepancies surface between the two translators regarding the phrasing of the title, instructions, and response key (options 2 and 4). Specifically, 14 items had disagreements: items 2, 3, 4, 5, 6, 7, 8, 10, 11, 12, 14, 15, 16, and 17. Only item 9 presented disagreement regarding content.

For the synthesis of the translated versions, the response key and four items (4, 9, 13, and 15) remained unchanged even after undergoing at least one translation. Modifications were made in the title, instructions, and the remaining 13 items. The pronoun “eu” (“I” in English) was added to items where the sentence structure in the first-person required a personal pronoun. Item 9 underwent redefinition by consensus.

In the back-translation stage, disagreements occurred between the two translators regarding the phrasing of the title, instructions, options 3 and 4 of the response key, and 12 items (3, 6, 7, 8, 9, 10, 11, 12, 13, 14, 16, and 17). Items 6, 7, 12, and 17 presented discrepancies in content.

The expert committee adjusted the title and response instructions, substituting the noun “indivíduo” with “pessoa.” The only modified response key was number 4. Nine items were preserved according to the synthesis version, while eight items (3, 4, 6, 7, 9, 10, 12, 16) were adjusted to meet the equivalence criteria. After incorporating the committee's changes and adjustments, the cross-culturally adapted version of the VENI was established (see Appendix A). All data recorded throughout these stages are presented in Chart 1.

**Chart 1:** The cross-cultural adaptation process of the VENI into Brazilian Portuguese

Original version	Translation	Synthesis	Back-translation	Committee review
Title				
Voice-related Experiences of Nonbinary Individuals – VENI	T1. Experiências vocais de indivíduos não binários - EVINB	CBPV. Experiências relacionadas a Voz de Indivíduos Não Binários - VENI-Br	BT1. Voice related experiences of non-binary individuals	CBPV. Experiências relacionadas a Voz de Pessoas Não Binárias - VENI-BrR
	T2. Experiências relacionadas a Voz de Indivíduos Não-Binários		BT2. Voice related experiences of non-binary individuals	
For each of the following statements, please circle the rating that fits best based on your experience as a nonbinary individual.	T1. Para cada uma das afirmações a seguir, circule a classificação que melhor se encaixa à sua experiência como indivíduo não binário.	CBPV. Para cada uma das afirmações a seguir, marque a resposta que melhor representa a sua experiência como um indivíduo não binário	BT1. For each of the statements below, mark the answer that best represents your experience as a nonbinary individual	CBPV. Para cada uma das afirmações a seguir, marque a resposta que melhor representa a sua experiência como uma pessoa não binária	
	T2. Para cada uma das afirmações a seguir, circule a resposta que melhor representa a sua experiência como um indivíduo não binário.		BT2. For each of the following statements, mark the answer that best represents your experience as a non-binary individual		
Response scale					
1 = never or rarely	T1. nunca ou raramente	CBPV. nunca ou raramente	BT1. Never or rarely	CBPV. nunca ou quase nunca	
	T2.nunca ou raramente		BT2. never or rarely		

2 = sometimes	T1. as vezes	CBPV. as vezes	BT1. sometimes	CBPV. as vezes	
	T2. algumas vezes		BT2. sometimes		
3 = often	T1. frequentemente	CBPV. frequentemente	BT1. frequently	CBPV. frequentemente	
	T2. frequentemente		BT2. often		
4 = usually or always	T1.usualmente ou sempre	CBPV. geralmente ou sempre	BT1. Generally or always	CBPV. quase sempre ou sempre	
	T2. geralmente ou sempre		BT2. usually or always		
Items					
1 – The quality of my voice varies throughout the day.	T1.A qualidade da minha voz varia ao longo do dia	CBPV. A qualidade da minha voz varia durante o dia	BT1. The quality of my voice varies during the day	CBPV. A qualidade da minha voz varia durante o dia	
	T2. A qualidade da minha voz varia ao longo do dia.		BT2. The quality of my voice varies during the day		
2 – It is difficult to control the pitch of my voice.	T1. É difícil controlar o tom da minha voz.	CBPV. É difícil controlar o tom (fino ou grosso) da minha voz	BT1. It is difficult to control the tone (high or low) of my voice	CBPV. É difícil controlar o tom (fino ou grosso) da minha voz	
	T2. Eu tenho dificuldade em controlar o tom da minha voz.		BT2. It is difficult to control the pitch (high or low) of my voice		
3 – Some emotions cause my pitch to change beyond my control.	T1. algumas emoções fazem o tom da minha voz mudar independente da minha vontade	CBPV. Algumas emoções mudam o tom (fino ou grosso) da minha voz sem que eu controle	BT1. Some emotions change the tone (high or low) of my voice without me controlling it	CBPV. Algumas emoções mudam o tom (fino ou grosso) da minha voz sem o meu controle	
	T2. Algumas emoções fazem com que o tom da minha voz mude além do meu controle.		BT2. Some emotions change the pitch (high or low) of my voice without my control		
4 – My voice changes unexpectedly depending on the situation.	T1. dependendo da situação, minha voz muda inesperadamente	CBPV. Minha voz muda inesperadamente dependendo da situação	BT1. My voice changes unexpectedly depending on the situation	CBPV. Minha voz muda do nada em algumas situações	
	T2. Minha voz muda inesperadamente dependendo da situação.		BT2. My voice changes unexpectedly depending on the situation		
5 – My pitch becomes less desirable by the end of the day.	T1. o tom da minha voz fica pior no final do dia	CBPV. O tom (fino ou grosso) da minha voz fica pior ao final do dia	BT1. The tone (high or low) of my voice gets worse at the end of the day	CBPV. O tom (fino ou grosso) da minha voz fica pior ao final do dia	
	T2.Meu pitch se torna menos desejável ao final do dia.		BT2. The pitch (high or low) of my voice gets worse at the end of the day		
6 – I experience strain when trying to make my voice sound like I want it to.	T1. sinto tensão quando tento fazer com que minha voz soe do jeito que eu quero	CBPV. Eu sinto tensão para fazer com que minha voz soe do jeito que eu quero	BT1. I feel tense about making my voice sound the way I want	CBPV. Eu fico tenso para fazer com que minha voz fique do jeito que eu quero	
	T2. Sinto que faço tensão para fazer minha voz soar como eu quero.		BT2. I feel tension to make my voice sound the way I want it		
7 – It takes a lot of effort and focus to sound the way I want to.	T1. Preciso me esforçar muito e ter foco para que minha voz soe do jeito que eu quero	CBPV. Eu faço esforço e preciso de foco para a minha voz ficar como eu quero	BT1. I make an effort and need to focus in order to get the voice I want	CBPV. Eu faço esforço e preciso prestar atenção para a minha voz ficar como eu quero	
	T2. Eu preciso de muito esforço e foco para a minha voz soar como eu quero.		BT2. I make an effort and need focus to my voice sound the way I want it.		
8 – I speak in public less often than I would like to because of my voice.	T1. Falo em público com uma frequência menor do que eu gostaria por causa da minha voz	CBPV. Eu falo em público menos do que eu gostaria por causa da minha voz	BT1. I speak less in public than I would like to because of my voice	CBPV. Eu falo em público menos do que eu gostaria por causa da minha voz	
	T2. Eu falo em público com menos frequência do que gostaria por causa da minha voz.		BT2. I speak in public less than I would like because of my voice		
9 – I suspect that people misgender me because of my voice.	T1. Eu acho que as pessoas não identificam o meu gênero por causa da minha voz	CBPV. Eu acho que as pessoas não identificam o meu gênero por causa da minha voz	BT1. I think that people do not identify my gender because of my voice	CBPV. Eu acho que não me identificam como uma pessoa não binária por causa da minha voz	
	T2. Eu suspeito que as pessoas me interpretam mal por causa da minha voz.		BT2. I think people don't identify my gender because of my voice		
10 – I speak to people close to me less often than I would like because of my voice.	T1. Eu falo menos do que eu gostaria com pessoas próximas a mim por causa da minha voz	CBPV. Eu falo com pessoas próximas menos do que eu gostaria por causa da minha voz	BT2. I speak less with people close to me than I would like to because of my voice	CBPV. Eu falo menos do que eu gostaria com pessoas próximas por causa da minha voz	
	T2. Eu falo com pessoas próximas a mim com menos frequência do que gostaria por causa da minha voz.		BT2. I speak to people close to me less than I would like because of my voice		
11 – I suspect that people react negatively to my voice.	T1. Eu acho que as pessoas reagem negativamente ao som da minha voz	CBPV. Eu acho que as pessoas reagem negativamente a minha voz	BT1. I think that people react negatively to my voice	CBPV. Eu acho que as pessoas reagem negativamente a minha voz	
	T2. Eu desconfio que as pessoas reagem negativamente à minha voz.		BT2. I think people react negatively to my voice		
12 – My voice gets in the way of me living as myself.	T1.Minha voz me impede de viver como eu sou	CBPV. Minha voz me limita viver como eu sou	BT1. My voice limits me to be the one I am	CBPV. Minha voz me atrapalha para eu viver como eu sou	
	T2. Minha voz me impede de viver como eu mesmo.		BT2. My voice limits me to live as I am		
13 – I dislike the sound of my voice.	T1. Eu não gosto do som da minha voz	CBPV. Eu não gosto do som da minha voz	BT1. I do not like the sound of my voice	CBPV. Eu não gosto do som da minha voz	
	T2. Eu não gosto do som da minha voz		BT2. I don't like the sound of my voice		
14 – I feel that others take me less seriously because of my voice.	T1. Acho que as pessoas não me levam muito a sério por causa da minha voz	CBPV. Eu sinto que os outros não me levam tão a sério por causa da minha voz	BT1. I feel that others do not take me so seriously because of my voice	CBPV. Eu sinto que os outros não me levam tão a sério por causa da minha voz	
	T2. Eu sinto que os outros me levam menos a sério por causa da minha voz.		BT2. I feel like others don't take me so seriously because of my voice		
15 – I feel that others think poorly of me because of my voice.	T1. Sinto que os outros pensam mal de mim por causa da minha voz	CBPV. Eu sinto que os outros pensam mal de mim por causa da minha voz	BT1. I feel that others think badly of me because of my voice	CBPV. Eu sinto que os outros pensam mal de mim por causa da minha voz	
	T2. Eu sinto que os outros pensam mal de mim por causa da minha voz.		BT2. I feel that others think badly of me because of my voice		
16 – I'm uncomfortable talking on the phone because I might be misgendered.	T1. Não me sinto à vontade para falar ao telefone porque meu gênero pode não ser reconhecido	CBPV. Eu não me sinto confortável ao telefone porque podem confundir meu gênero	BT1. I do not feel comfortable on the phone because people might confuse my gender	CBPV. Eu me sinto desconfortável ao telefone porque podem me confundir	
	T2. Eu não me sinto à vontade para falar ao telefone porque posso ser percebido com um gênero errado.		BT2. I don't feel comfortable on the phone because they can confuse my gender		
17 - I worry about how strangers perceive my voice.	T1. Eu me preocupo com a maneira como estranhos percebem a minha voz	CBPV. Eu me preocupo como as pessoas estranhas percebem a minha voz	BT1. I worry about how unfamiliar people perceive my voice	CBPV. Eu me preocupo como as pessoas estranhas percebem a minha voz	
	T2. Eu me preocupo em como as pessoas estranhas percebem a minha voz.		BT2. I worry about how strangers perceive my voice		

Caption: T1 = translator 1 English-Portuguese; T2 = translator 2 English-Portuguese; BT1 = back-translator 1 Portuguese-English; BT2 = back-translator 2 Portuguese-English; CBPV = consensus of the Brazilian Portuguese version

The proportion of participants who marked items 5, 8, 9, 10, 11, 12, 13, 14, 15, and 16 as non-applicable was significantly lower than those who selected one of the instrument's four standard response options. No participant chose the non-applicable option for items 1, 2, 3, 4, 6, 7, and 17. Table 1 shows the comparison of the proportion of responses. 

**Table 1:** Analysis of the comparison of the proportion of non-applicable responses and VENI response scale for each item of the instrument by non-binary persons

**Item**	**options 1-4**		**NA**		**p-value**
	**n**	**%**	**n**	**%**	
1	21	100.00	0	0.00	
2	21	100.00	0	0.00	
3	21	100.00	0	0.00	
4	21	100.00	0	0.00	
5	20	95.24	1	4.76	<0.001
6	21	100.00	0	0.00	
7	21	100.00	0	0.00	
8	19	90.48	2	9.52	<0.001
9	17	80.95	4	19.05	0.005
10	18	85.71	3	14.29	0.001
11	17	80.95	4	19.05	0.005
12	19	90.48	2	9.52	<0.001
13	19	90.48	2	9.52	<0.001
14	19	90.48	2	9.52	<0.001
15	19	90.48	2	9.52	<0.001
16	20	95.24	1	4.76	<0.001
17	21	100.00	0	0.00	

Binomial Test

Caption: n = absolute frequency; % = relative frequency; NA = not applicable

## DISCUSSION

Nonbinary individuals increasingly seek to voice and communication training to align their voices with their gender identity. Therefore, it is essential to develop specific SLP interventions for this population. Engaging in gender affirmation can enhance self-confidence, resilience, social relationships, and professional connections^(5)^, thus, gender affirming training can improve these individuals' quality of life. However, nonbinary individuals presents unique voice and communication needs^(6)^. Therefore, the intervention must be tailored-made and specific assessment instruments need to be reliable to better understand these individuals' perceptions of their communicative and vocal experiences, improving SLP services^(1,2)^. Hence, the VENI cross-cultural adaptation to Brazilian Portuguese (BP) is imperative.

The disagreements observed during the translation, back-translation and expert committee review regarding the form and content of items, response key, instructions, and title underscore the importance of following structured steps in the cross-cultural adaptation of an instrument. These outcomes emphasize that literal translation, which might overlook cultural nuances, is not advisable^(14)^.

Semantic equivalence necessitated adjustments to ensure that items 9, 12, and 16 in BP maintained the same meaning as the original version. For cultural equivalence, grammatical modifications were made to items 3, 4, 6, 7, 9, and 16 to reflect Brazilian culture^(15)^. Achieving conceptual equivalence involved replacing the term “indivíduos não binários” with “pessoas não binárias.” In Brazilian Portuguese, “indivíduo” (similar to individual) is a masculine singular noun, often emphasizing a person's singularity and uniqueness. On the other hand, “pessoa” (similar to person) is a gender-variable noun that emphasizes group belonging^(12)^. No adjustments were necessary to achieve idiomatic, experiential, and operational equivalences.

During the pre-test phase, the significantly higher proportion of standard response key selection indicated that all items were understood and pertinent to the vocal experiences of nonbinary individuals. Only three items (9, 10, and 11) showed non-applicable responses above 10%, but they remained statistically significant^(16)^.

These items underwent consensus-driven adjustments at almost every stage once the initial translation did not fully align with the original English version. Items 9 (“Eu acho que não me identificam como uma pessoa não binária por causa da minha voz”) and 11 (“Eu acho que as pessoas reagem negativamente a minha voz”) explore the nonbinary person's perception of others' opinions and reactions towards them. Item 10 (“Eu falo menos do que eu gostaria com pessoas próximas por causa da minha voz”) reflects a subjective experience, dependent on the person’s communicative profile and not solely on their gender. These three items reference subjective experiences that are not necessarily shared or observed by all nonbinary individuals, which probably led to the non-applicable option selection. However, the Brazilian culture-adapted version captures the instrument's core content.

Consequently, all items were considered understandable and related to the nonbinary population. No further adjustments were required following the application of the cross-culturally adapted version to this target population. The cross-cultural adaptation conducted in this study was successful, rendering the VENI applicable and understandable in BP. This adaptation marks the commencement of refining instruments targeted at clinical and research services that focus on the voice and communication of nonbinary individuals in Brazil. The instrument should undergo validation to confirm its structure, validity, and reliability in BP.

## CONCLUSION

The VENI was successfully adapted to Brazilian Portuguese, resulting in the Brazilian version known as “Experiências relacionadas à Voz de Pessoas Não Binárias - VENI-Br.” The responses collected during the pre-test phase confirm the success of the cross-cultural adaptation process for the VENI.

## Appendix A Cross-culturally adapted version of the Voice-related Experiences of Nonbinary Individuals – VENI

Experiências relacionadas a Voz de Pessoas Não Binárias - VENI-Br

Chave de resposta:

1 = nunca ou quase nunca

2 = as vezes

3 = frequentemente

4 = quase sempre ou sempre

**Table:** Para cada uma das afirmações a seguir, marque a resposta que melhor representa a sua experiência como uma pessoa não binária

1. A qualidade da minha voz varia durante o dia.	**1**	**2**	**3**	**4**
2. É difícil controlar o tom (fino ou grosso) da minha voz.	**1**	**2**	**3**	**4**
3. Algumas emoções mudam o tom (fino ou grosso) da minha voz sem o meu controle.	**1**	**2**	**3**	**4**
4. Minha voz muda do nada em algumas situações.	**1**	**2**	**3**	**4**
5. O tom (fino ou grosso) da minha voz fica pior ao final do dia.	**1**	**2**	**3**	**4**
6. Eu sinto tensão para fazer com que minha voz fique do jeito que eu quero.	**1**	**2**	**3**	**4**
7. Eu faço esforço e preciso prestar atenção para a minha voz ficar como eu quero.	**1**	**2**	**3**	**4**
8. Eu falo em público menos do que eu gostaria por causa da minha voz.	**1**	**2**	**3**	**4**
9. Eu acho que não me identificam como uma pessoa não binária por causa da minha voz.	**1**	**2**	**3**	**4**
10. Eu falo menos do que eu gostaria com pessoas próximas por causa da minha voz.	**1**	**2**	**3**	**4**
11. Eu acho que as pessoas reagem negativamente a minha voz.	**1**	**2**	**3**	**4**
12. Minha voz me atrapalha para eu viver como eu sou.	**1**	**2**	**3**	**4**
13. Eu não gosto do som da minha voz.	**1**	**2**	**3**	**4**
14. Eu sinto que os outros não me levam tão a sério por causa da minha voz.	**1**	**2**	**3**	**4**
15. Eu sinto que os outros pensam mal de mim por causa da minha voz.	**1**	**2**	**3**	**4**
16. Eu me sinto desconfortável ao telefone porque podem me confundir.	**1**	**2**	**3**	**4**
17. Eu me preocupo como as pessoas estranhas percebem a minha voz.	**1**	**2**	**3**	**4**

Itens Físicos: 1, 2, 3, 4, 5, 6, 7

Itens Funcionais: 8, 9, 10, 11, 12, 14, 15, 16

Itens Emocionais: 13, 17
