# Supplementary material for: Cross-cultural adaptation of the Voice-related Experiences of Nonbinary Individuals - VENI to Brazilian Portuguese
Source: Codas. 2024 May 27;36(3):e20230170. doi: 10.1590/2317-1782/20242023170en (PMC11166032; doi:10.1590/2317-1782/20242023170en)
Supplement: Translation

# Translation: Adaptação transcultural do Voice-related Experiences of Nonbinary Individuals – VENI para o português brasileiro

**DOI:** 10.1590/2317-1782/20242023170pt

**Authors:** Isabela dos Santos, Mara Behlau, Grace Shefcik, Pei-tzu Tsai, Vanessa Veis Ribeiro

**Affiliations:** 1 Curso de Especialização em Voz – CECEV, Centro de Estudos da Voz – CEV - São Paulo (SP), Brasil.; 2 Programa Associado de Pós-graduação em Fonoaudiologia – PPGFON, Universidade Federal da Paraíba – UFPB, Universidade Estadual de Ciências da Saúde de Alagoas, Universidade Estadual de Ciências da Saúde de Alagoas – UNCISAL - João Pessoa (PB), Brasil.; Universidade Federal do Rio Grande do Norte – UFRN,; 3 Department of Communicative Disorders and Sciences, San José State University - San Jose (CA), USA.; 4 Curso de Fonoaudiologia, Faculdade de Ceilândia, Universidade de Brasília – UNB - Brasília (DF), Brasil.

**Keywords:** Voz, Protocolos Clínicos, Autoavaliação, Qualidade de Vida, Identidade de Gênero, Fonoaudiologia

## Abstract

**Objetivo:**

Traduzir e adaptar transculturalmente o Voice-related Experiences of Nonbinary Individuals – VENI para o português brasileiro.

**Método:**

Os procedimentos de adaptação transcultural foram baseados na combinação das recomendações e diretrizes da World Health Organization (WHO) Guidelines on Translation com o COnsensus-based Standards for the selection of health Measurement INstruments (COSMIN). Foram realizadas cinco etapas: a) tradução do instrumento para o Português Brasileiro (PB) por um tradutor especialista no construto e um não-especialista, nativos do PB e fluentes em inglês; b) elaboração da síntese das duas traduções por consenso; c) retrotradução por um tradutor especialista no construto e um não-especialista, nativos do inglês e fluentes em PB; d) análise de um comitê formado por cinco fonoaudiólogos especialistas em voz e elaboração da versão final; e) pré-teste com 21 pessoas da população-alvo, aplicado virtualmente.

**Resultados:**

Na tradução houve discordância no título, instruções, chave de resposta e em 15 itens. Na retrotradução, houve discordância quanto à forma em 12 itens e ao conteúdo em 4 itens. A análise do comitê de especialistas indicou mudanças no título, instruções de resposta, uma opção da chave de resposta, e em oito itens, para atender aos critérios de equivalência. No pré-teste houve proporção significativamente maior de respostas habituais do instrumento quando comparadas com a opção não-aplicável, usada regularmente nas adaptações de instrumentos.

**Conclusão:**

A adaptação transcultural para o português brasileiro do VENI foi bem sucedida e resultou na versão denominada “Experiências relacionadas a Voz de Pessoas Não Binárias - VENI-Br”.

## INTRODUÇÃO

A qualidade vocal reflete aspectos da fonte do som (laringe) e dos filtros de ressonância (trato vocal), incluindo também: aspectos neuromusculares de produção e controle vocal; monitoramento auditivo; traços de personalidade; preferências pessoais e elementos culturais^(1)^. A voz tem um papel importante na representatividade do indivíduo como um ser único, com suas particularidades, além de ser central na socialização, identidade, experiências comunicativas e cultura; desta forma, é fundamental entender as necessidades e preferências vocais de cada pessoa^(1,2)^.

Especificamente na população transgênero, a voz deve refletir a identidade de gênero, com grande influência nas questões sociais de inclusão, sobrevivência e qualidade de vida^(3)^. A identidade de gênero é um termo que indica o senso individual que uma pessoa possui de seu próprio gênero, uma vez que é experimentado em particular, na autoconsciência de estar dentro ou fora de um gênero; essa identidade pode ser masculina, feminina, não binária ou fluida^(4,5)^. Sexo e gênero são comumente apresentados como equivalentes, mas representam conceitos distintos. O sexo está relacionado a aspectos biológicos, como cromossomos, hormônios e órgãos reprodutivos, caracterizados como imutáveis e binários (masculino ou feminino). Convencionalmente, a identificação sexual baseia-se na configuração cromossômica XX para mulheres e XY para homens. Já o gênero incorpora traços e identidades que são construídos com base em normas sociais e culturais. Além disso, no conceito de gênero, a autoidentificação tem importância central. Todos esses aspectos permitem apreciar um espectro amplo que possibilita categorizações que excedem à proposta binária de sexo e gênero em masculino e feminino^(4)^.

Alguns indivíduos se identificam exclusivamente com um gênero binário atribuído ao seu nascimento e que está associado à sua genitália ao nascer; na concepção binária, é referida como homem ou mulher cisgênero. Quando não há identificação com o gênero que lhes foi associado ao nascimento, as pessoas são nomeadas homem ou mulher transgênero. Contudo, a classificação binária é apenas uma possibilidade e há um terceiro grupo de pessoas que a considera rígida e compartimentalizada, que se identifica de forma fluida com um dos gêneros binários, ambos ou nenhum. Pessoas não binárias representam uma demanda crescente da clínica fonoaudiológica^(4-6)^.

A importância da voz e da comunicação como identidade de gênero para pessoas de gêneros diversos justifica o crescimento da busca pela intervenção fonoaudiológica para afirmação de gênero por meio da comunicação, com foco especial sobre a voz. Diversos ajustes podem ser explorados no trabalho com essa população, como qualidade vocal neutra, variação da frequência fundamental, mudanças na ressonância, classificação, e aspectos relacionados à comunicação não verbal^(7)^.

As pessoas não binárias têm demandas comunicativas e vocais específicas, diferentes dos binários, o que merece estudo para melhor compreensão dessa realidade, tendo em vista um trabalho fonoaudiológico adequado. Essas pessoas podem buscar vozes femininas, masculinas, neutras ou expansivas em termos de gênero^(6,8)^. A autopercepção das características vocais é subjetiva, baseada em comparações, envolvendo aspectos emocionais, de personalidade e experiências. A autoavaliação vocal tem grande valor clínico, pois capta a percepção do cliente em relação a sua voz e auxilia no direcionamento do trabalho fonoaudiológico^(1,2)^.

Para compreender melhor as experiências comunicativas e a autoavaliação vocal das pessoas não binárias são necessários instrumentos específicos de avaliação^(9,10)^. Atualmente, o único instrumento validado que é específico para essa população é o *Voice-related Experiences of Nonbinary Individuals* (VENI). O VENI avalia as experiências relacionadas a voz deste grupo e contribui para o desenvolvimento de serviços de comunicação centrados no cliente e que afirmam o gênero. É composto por 17 itens que buscam, com as pontuações quantitativas, traçar estratégias, acompanhar o progresso e documentar as mudanças na autopercepção vocal^(6)^. Esse é o único instrumento especificamente desenvolvido para essa população, elaborado e validado em inglês. Dessa forma, faz-se necessário realizar a adaptação transcultural e validação para que possa ser utilizado em outro idioma e cultura.

A adaptação transcultural é a etapa inicial do processo de validação de um instrumento e tem como objetivo fazer com que o conteúdo e propósito dos itens originais correspondam ao contexto linguístico e cultural abordados^(9-12)^. A adaptação transcultural do VENI para o português brasileiro contribuirá para o acolhimento das preocupações, experiências, percepções e voz alvo de pessoas não binárias no país, que procuram a clínica vocal, além de facilitar o planejamento da intervenção fonoaudiológica e a obtenção de melhores resultados por essa população^(6,8)^.

Sendo assim, o objetivo desse estudo foi realizar a adaptação transcultural do VENI para o português brasileiro.

## MÉTODO

Trata-se de um estudo de delineamento transversal e natureza metodológica, aprovado pelo Comitê de Ética em Pesquisa da Universidade de Brasília (parecer 5.845.012). A pesquisa seguiu às orientações éticas estabelecidas pela Resolução nº 466/2012 e demais normativas legais em vigor no Brasil. Todos os participantes assinaram o Termo de Consentimento Livre e Esclarecido (TCLE).

O VENI foi desenvolvido na língua inglesa. Ele é composto por 17 itens que se dividem entre três fatores: sete itens associados às questões físicas (itens 1 a 7), oito itens associados às questões funcionais (itens 8,9,10,11,12,14,15 e 16) e dois itens associados às emocionais (itens 13 e 17). Cada item oferece uma chave de resposta com quatro opções: 1 *= never or rarely;* 2 *= sometimes;* 3 *= often e* 4 *= usually or always*. Os itens em seus respectivos fatores são pontuados por somatório simples^(6)^.

O processo de adaptação transcultural do VENI para o português brasileiro foi embasado na combinação das recomendações e diretrizes da *World Health Organization* (WHO) *Guidelines on Translation* ^(9)^ com o *COnsensus-based Standards for the selection of health Measurement INstruments* (COSMIN)^(10)^.

Para compor o grupo de juízes (tradução, retrotradução e comitê de especialistas) e de participantes do pré-teste foram seguidas as recomendações de quantitativo e qualificação recomendados pelo COSMIN^(10).^ Todas as etapas foram realizadas de forma virtual.

O teste foi traduzido de forma individual por dois juízes, sendo um fonoaudiólogo especialista no construto e um não-especialista, nativos no idioma alvo (PB) e fluentes no idioma e cultura fonte (inglês). O processo considerou a equivalência conceitual e evitou a tradução literal. Os juízes traduziram independentemente o título, as instruções, a chave de resposta e os itens.

Os autores elaboraram uma versão de síntese por consenso das duas traduções para o título, instruções, chave de resposta e itens, e resolveram as discordâncias entre os juízes.

Essa versão em PB foi retrotraduzida para o inglês por dois juízes, sendo um fonoaudiólogo especialista no construto e um não-especialista, fluentes no idioma e cultura alvo (português) e nativos do idioma fonte (inglês).

Um comitê formado por cinco fonoaudiólogos (tradutores, autores e especialistas) realizou a análise da versão final, por consenso, em uma reunião. O grupo comparou a retrotradução ao instrumento original e verificou a necessidade de mudanças para alcançar as seguintes equivalências:

- Equivalência semântica: observaram se as palavras da versão final tinham o mesmo significado da versão original;
- Equivalência conceitual: identificaram se havia palavras ou expressões que possuíam significado conceitual diferente entre as culturas, e substituíram tal palavra ou expressão;
- Equivalência idiomática: observaram se havia necessidade de reformular alguma expressão equivalente na língua-alvo quando havia expressões idiomáticas coloquiais difíceis de traduzir;
- Equivalência experiencial: observaram se havia necessidade de substituir algum item original por um item semelhante que existisse na cultura-alvo;
- Equivalência cultural: realizaram ajustes ortográficos ou gramaticais nos itens;
- Equivalência operacional: observaram se havia necessidade de modificar os procedimentos inerentes à aplicação do instrumento.

Todas as etapas foram documentadas em uma tabela geral que foi alimentada conforme eram concluídas.

A versão final do instrumento foi submetida ao pré-teste, que consiste em uma aplicação da versão transculturalmente adaptada do instrumento junto a população-alvo. Nessa fase foi acrescida a opção de resposta não aplicável (N/A) à chave de resposta do instrumento. Os participantes foram orientados a marcar esta opção caso o item não pudesse ser compreendido ou não se aplicasse na cultura-alvo. Caso algum item apresentasse problemas, seria revisado.

Os critérios de elegibilidade para participar do pré-teste foram: pessoas que se identificassem como não binárias, com idade entre 18 anos e 65 anos. Foram excluídas pessoas que autorreferissem problemas cognitivos que dificultassem a compreensão do instrumento.

O cálculo do tamanho da amostra foi realizado por meio do teste estatístico de intervalo de uma proporção. Os parâmetros adotados para o teste foram probabilidade de erro tipo I (α) de 5%, probabilidade de erro tipo II (β) de 10% e poder do teste (K) de 90%. Para o cálculo foi utilizada a estimativa de 1,19% de pessoas não binárias no Brasil^(13)^. O tamanho da amostra calculado foi de 19 participantes. Utilizou-se o software *Statistica* versão 11.0. Dessa forma, foram selecionados para participar do pré-teste 21 pessoas não binárias, com média de idade de 30,71 anos (DP: 8,66).

Os participantes foram recrutados de maneira virtual, nas redes sociais e digitais, por meio da veiculação de um *folder* com um *link* de acesso para a pesquisa, ao clicar no *link* os participantes eram encaminhados para a plataforma *Google Forms*. Aqueles que atenderam aos critérios de elegibilidade e concordaram com o TCLE tiveram acesso aos instrumentos de coleta de dados, que se referiram a um questionário sobre dados de identificação dos participantes e ao VENI.

Os dados do pré-teste foram analisados com o *software* SPSS 25.0 por meio do Teste Qui-Quadrado para uma amostra. Foi comparada a proporção de respostas não-aplicável e a proporção de respostas habituais da chave de resposta do instrumento (1, 2, 3 ou 4) para cada item com o Teste Binomial.

## RESULTADOS

Na etapa inicial da tradução, houve discordância na forma do título, instruções e chave de resposta (opções 2 e 4) entre os dois tradutores. Nos itens, houve discordância na forma de 14 itens, sendo estes 2, 3, 4, 5, 6, 7, 8, 10, 11, 12, 14, 15, 16 e 17, porém, apenas no item 9 houve discordância quanto ao conteúdo.

Para a síntese das versões traduzidas, a chave de resposta e quatro itens (4, 9, 13 e 15) foram mantidos de acordo com pelo menos uma das traduções realizadas. Título, instruções e os 13 itens restantes foram modificados na forma apresentada. Inclui-se o pronome “eu” nos itens em que o início das frases exigia um pronome pessoal, devido à estrutura da frase estar na primeira pessoa do singular. O item 9 foi redefinido por consenso.

Na retrotradução, houve discordância entre os dois tradutores quanto à forma para o título, instruções, opções 3 e 4 da chave de resposta e para 12 itens (3, 6, 7, 8, 9, 10, 11, 12, 13, 14, 16 e 17). Os itens 6, 7, 12 e 17 indicaram discordância no conteúdo.

O comitê de especialistas realizou ajustes no título e nas instruções de resposta, alterando o substantivo “indivíduo” para “pessoa”. A única opção da chave de resposta alterada foi a 4. Mantiveram-se nove itens de acordo com a versão de síntese, e oito (3, 4, 6, 7, 9, 10, 12, 16) foram ajustados para atender aos critérios de equivalência. Após concluir a etapa com base nas alterações e ajustes do comitê, estabelece-se a versão transculturalmente adaptada do VENI (Apêndice A). Todos os dados descritos ao longo das etapas podem ser observados no Quadro 1.

**Quadro 1:** Processo de adaptação transcultural do VENI para o português brasileiro

Versão Original	Tradução	Síntese	Retrotradução	Consenso
Título				
Voice-related Experiences of Nonbinary Individuals – VENI	T1. Experiências vocais de indivíduos não binários - EVINB	VCP. Experiências relacionadas a Voz de Indivíduos Não Binários - VENI-Br	R1. Voice related experiences of non-binary individuals	VCP. Experiências relacionadas a Voz de Pessoas Não Binárias - VENI-BrR
	T2. Experiências relacionadas a Voz de Indivíduos Não-Binários		R2. Voice related experiences of non-binary individuals	
For each of the following statements, please circle the rating that fits best based on your experience as a nonbinary individual.	T1. Para cada uma das afirmações a seguir, circule a classificação que melhor se encaixa à sua experiência como indivíduo não binário.	VCP. Para cada uma das afirmações a seguir, marque a resposta que melhor representa a sua experiência como um indivíduo não binário	R1. For each of the statements below, mark the answer that best represents your experience as a nonbinary individual	VCP. Para cada uma das afirmações a seguir, marque a resposta que melhor representa a sua experiência como uma pessoa não binária
	T2. Para cada uma das afirmações a seguir, circule a resposta que melhor representa a sua experiência como um indivíduo não binário.		R2. For each of the following statements, mark the answer that best represents your experience as a non-binary individual	
Chave de resposta				
1 = never or rarely	T1. nunca ou raramente	VCP. nunca ou raramente	R1. Never or rarely	VCP. nunca ou quase nunca
	T2.nunca ou raramente		R2. never or rarely	
2 = sometimes	T1. as vezes	VCP. as vezes	R1. sometimes	VCP. as vezes
	T2. algumas vezes		R2. sometimes	
3 = often	T1. frequentemente	VCP. frequentemente	R1. frequently	VCP. frequentemente
	T2. frequentemente		R2. often	
4 = usually or always	T1.usualmente ou sempre	VCP. geralmente ou sempre	R1. Generally or always	VCP. quase sempre ou sempre
	T2. geralmente ou sempre		R2. usually or always	
Itens				
1 – The quality of my voice varies throughout the day.	T1. A qualidade da minha voz varia ao longo do dia	VCP. A qualidade da minha voz varia durante o dia	R1. The quality of my voice varies during the day	VCP. A qualidade da minha voz varia durante o dia
	T2. A qualidade da minha voz varia ao longo do dia.		R2. The quality of my voice varies during the day	
2 – It is difficult to control the pitch of my voice.	T1. É difícil controlar o tom da minha voz.	VCP. É difícil controlar o tom (fino ou grosso) da minha voz	R1. It is difficult to control the tone (high or low) of my voice	VCP. É difícil controlar o tom (fino ou grosso) da minha voz
	T2. Eu tenho dificuldade em controlar o tom da minha voz.		R2. It is difficult to control the pitch (high or low) of my voice	
3 – Some emotions cause my pitch to change beyond my control.	T1. algumas emoções fazem o tom da minha voz mudar independente da minha vontade	VCP. Algumas emoções mudam o tom (fino ou grosso) da minha voz sem que eu controle	R1. Some emotions change the tone (high or low) of my voice without me controlling it	VCP. Algumas emoções mudam o tom (fino ou grosso) da minha voz sem o meu controle
	T2. Algumas emoções fazem com que o tom da minha voz mude além do meu controle.		R2. Some emotions change the pitch (high or low) of my voice without my control	
4 – My voice changes unexpectedly depending on the situation.	T1. dependendo da situação, minha voz muda inesperadamente	VCP. Minha voz muda inesperadamente dependendo da situação	R1. My voice changes unexpectedly depending on the situation	VCP. Minha voz muda do nada em algumas situações
	T2. Minha voz muda inesperadamente dependendo da situação.		R2. My voice changes unexpectedly depending on the situation	
5 – My pitch becomes less desirable by the end of the day.	T1. o tom da minha voz fica pior no final do dia	VCP. O tom (fino ou grosso) da minha voz fica pior ao final do dia	R1. The tone (high or low) of my voice gets worse at the end of the day	VCP. O tom (fino ou grosso) da minha voz fica pior ao final do dia
	T2.Meu pitch se torna menos desejável ao final do dia.		R2.The pitch (high or low) of my voice gets worse at the end of the day	
6 – I experience strain when trying to make my voice sound like I want it to.	T1. sinto tensão quando tento fazer com que minha voz soe do jeito que eu quero	VCP. Eu sinto tensão para fazer com que minha voz soe do jeito que eu quero	R1. I feel tense about making my voice sound the way I want	VCP. Eu fico tenso para fazer com que minha voz fique do jeito que eu quero
	T2. Sinto que faço tensão para fazer minha voz soar como eu quero.		R2. I feel tension to make my voice sound the way I want it	
7 – It takes a lot of effort and focus to sound the way I want to.	T1. Preciso me esforçar muito e ter foco para que minha voz soe do jeito que eu quero	VCP. Eu faço esforço e preciso de foco para a minha voz ficar como eu quero	R1. I make an effort and need to focus in order to get the voice I want	VCP. Eu faço esforço e preciso prestar atenção para a minha voz ficar como eu quero
	T2. Eu preciso de muito esforço e foco para a minha voz soar como eu quero.		R2. I make an effort and need focus to my voice sound the way I want it.	
8 – I speak in public less often than I would like to because of my voice.	T1. Falo em público com uma frequência menor do que eu gostaria por causa da minha voz	VCP. Eu falo em público menos do que eu gostaria por causa da minha voz	R1. I speak less in public than I would like to because of my voice	VCP. Eu falo em público menos do que eu gostaria por causa da minha voz
	T2. Eu falo em público com menos frequência do que gostaria por causa da minha voz.		R2. I speak in public less than I would like because of my voice	
9 – I suspect that people misgender me because of my voice.	T1. Eu acho que as pessoas não identificam o meu gênero por causa da minha voz	VCP. Eu acho que as pessoas não identificam o meu gênero por causa da minha voz	R1. I think that people do not identify my gender because of my voice	VCP. Eu acho que não me identificam como uma pessoa não binária por causa da minha voz
	T2. Eu suspeito que as pessoas me interpretam mal por causa da minha voz.		R2. I think people don't identify my gender because of my voice	
10 – I speak to people close to me less often than I would like because of my voice.	T1. Eu falo menos do que eu gostaria com pessoas próximas a mim por causa da minha voz	VCP. Eu falo com pessoas próximas menos do que eu gostaria por causa da minha voz	R2. I speak less with people close to me than I would like to because of my voice	VCP. Eu falo menos do que eu gostaria com pessoas próximas por causa da minha voz
	T2. Eu falo com pessoas próximas a mim com menos frequência do que gostaria por causa da minha voz.		R2. I speak to people close to me less than I would like because of my voice	
11 – I suspect that people react negatively to my voice.	T1. Eu acho que as pessoas reagem negativamente ao som da minha voz	VCP. Eu acho que as pessoas reagem negativamente a minha voz	R1. I think that people react negatively to my voice	VCP. Eu acho que as pessoas reagem negativamente a minha voz
	T2. Eu desconfio que as pessoas reagem negativamente à minha voz.		R2. I think people react negatively to my voice	
12 – My voice gets in the way of me living as myself.	T1.Minha voz me impede de viver como eu sou	VCP. Minha voz me limita viver como eu sou	R1. My voice limits me to be the one I am	VCP. Minha voz me atrapalha para eu viver como eu sou
	T2. Minha voz me impede de viver como eu mesmo.		R2. My voice limits me to live as I am	
13 – I dislike the sound of my voice.	T1. Eu não gosto do som da minha voz	VCP. Eu não gosto do som da minha voz	R1. I do not like the sound of my voice	VCP. Eu não gosto do som da minha voz
	T2. Eu não gosto do som da minha voz		R2. I don't like the sound of my voice	
14 – I feel that others take me less seriously because of my voice.	T1. Acho que as pessoas não me levam muito a sério por causa da minha voz	VCP. Eu sinto que os outros não me levam tão a sério por causa da minha voz	R1. I feel that others do not take me so seriously because of my voice	VCP. Eu sinto que os outros não me levam tão a sério por causa da minha voz
	T2. Eu sinto que os outros me levam menos a sério por causa da minha voz.		R2. I feel like others don't take me so seriously because of my voice	
15 – I feel that others think poorly of me because of my voice.	T1. Sinto que os outros pensam mal de mim por causa da minha voz	VCP. Eu sinto que os outros pensam mal de mim por causa da minha voz	R1. I feel that others think badly of me because of my voice	VCP. Eu sinto que os outros pensam mal de mim por causa da minha voz
	T2. Eu sinto que os outros pensam mal de mim por causa da minha voz.		R2. I feel that others think badly of me because of my voice	
16 – I’m uncomfortable talking on the phone because I might be misgendered.	T1. Não me sinto à vontade para falar ao telefone porque meu gênero pode não ser reconhecido	VCP. Eu não me sinto confortável ao telefone porque podem confundir meu gênero	R1. I do not feel comfortable on the phone because people might confuse my gender	VCP. Eu me sinto desconfortável ao telefone porque podem me confundir
	T2. Eu não me sinto à vontade para falar ao telefone porque posso ser percebido com um gênero errado.		R2. I don't feel comfortable on the phone because they can confuse my gender	
17 - I worry about how strangers perceive my voice.	T1. Eu me preocupo com a maneira como estranhos percebem a minha voz	VCP. Eu me preocupo como as pessoas estranhas percebem a minha voz	R1. I worry about how unfamiliar people perceive my voice	VCP. Eu me preocupo como as pessoas estranhas percebem a minha voz
	T2. Eu me preocupo em como as pessoas estranhas percebem a minha voz.		R2. I worry about how strangers perceive my voice	

Legenda: T1 = tradutor 1 inglês-português; T2 = tradutor 2 inglês-português; R1 = retrotradutor 1 português-inglês; R2 = retrotradutor 2 português-inglês; VCP = versão em português do consenso

A proporção de participantes que responderam não aplicável aos itens 5, 8, 9, 10, 11, 12, 13, 14, 15 e 16 foi significativamente menor que a proporção de participantes que respondem a uma das quatro opções habituais da chave de resposta do instrumento. Para os itens 1, 2, 3, 4, 6, 7 e 17, nenhum participante respondeu não aplicável. A Tabela 1 contempla a análise da comparação de proporção das respostas.

**Tabela 1:** Análise da comparação de proporção de respostas não-aplicáveis e de respostas da chave de resposta do VENI para cada item do instrumento por pessoas não binárias

**Item**	**1 a 4**		**NA**		**p-valor**
	**n**	**%**	**n**	**%**	
1	21	100,00	0	0,00	
2	21	100,00	0	0,00	
3	21	100,00	0	0,00	
4	21	100,00	0	0,00	
5	20	95,24	1	4,76	<0,001
6	21	100,00	0	0,00	
7	21	100,00	0	0,00	
8	19	90,48	2	9,52	<0,001
9	17	80,95	4	19,05	0,005
10	18	85,71	3	14,29	0,001
11	17	80,95	4	19,05	0,005
12	19	90,48	2	9,52	<0,001
13	19	90,48	2	9,52	<0,001
14	19	90,48	2	9,52	<0,001
15	19	90,48	2	9,52	<0,001
16	20	95,24	1	4,76	<0,001
17	21	100,00	0	0,00	

Teste Binomial

Legenda: n = frequência absoluta; % = frequência relativa; NA = não aplicável

## DISCUSSÃO

Pessoas não binárias têm um lugar cada vez mais frequente na fonoaudiologia, buscando uma voz que reflita sua identidade de gênero. Deve-se desenvolver ações fonoaudiológicas específicas para essa população. Trabalhar afirmação de gênero, pode melhorar autoconfiança, resiliência, conexões sociais e conexões laborais^(5)^, tornando o trabalho fonoaudiológico importante para a qualidade de vida dessas pessoas. Porém, trata-se de uma população com demandas de voz e comunicação específicas^(6)^. Logo, faz-se necessário que o atendimento fonoaudiológico possua instrumentos particulares que permitam compreender a percepção dessas pessoas quanto as experiências comunicativas e vocais, e assim, aprimorar o direcionamento do atendimento^(1,2)^. Diante disso, torna-se importante adaptar transculturalmente para o PB o VENI.

As discordâncias na forma e conteúdo dos itens, chave de resposta, instruções e título, encontradas durante o processo de tradução, retrotradução e a revisão do comitê de especialistas no VENI, reforçam o quão importante é seguir etapas bem estruturadas no processo de adaptação transcultural de um instrumento. Esses dados reforçam que uma tradução literal não contempla aspectos culturais e não é recomendada^(14)^.

A equivalência semântica indicou ajustes para que os itens 9, 12 e 16 em PB tivessem o mesmo significado da versão original. Para a equivalência cultural, foram realizadas adaptações gramaticais nos itens 3, 4, 6, 7, 9 e 16 contextualizando à cultura brasileira^(15)^. Para obter equivalência conceitual, optou-se pela troca do termo “indivíduos não binários” para “pessoas não binárias”, pois no português brasileiro, “indivíduo” é um substantivo masculino singular que frequentemente se concentra na singularidade e individualidade de uma pessoa, enquanto o termo “pessoa” é um substantivo comum que varia gênero e enfatiza o pertencimento a um grupo maior^(12)^. Não houve necessidade de ajustes no instrumento para obtenção das equivalências conceitual, experiencial e operacional.

Na etapa do pré-teste, a proporção significativamente maior da chave de resposta habitual mostrou que todos os itens foram compreendidos e se aplicam às experiências vocais das pessoas não binárias. Apenas três itens (9, 10 e 11) apresentaram proporção de não-aplicável acima de 10%, porém mantendo-se estatisticamente significativos^(16)^.

Esses itens sofrem ajustes em consenso, em quase todas as etapas pelo fato de a tradução apresentada não ser totalmente equivalente ao original em inglês. Os itens 9 (“Eu acho que não me identificam como uma pessoa não binária por causa da minha voz”) e 11 (“Eu acho que as pessoas reagem negativamente a minha voz”) tratam-se da opinião da pessoa não binária sobre o que ela acha da opinião e reação dos outros com ela. Já o item 10 (“Eu falo menos do que eu gostaria com pessoas próximas por causa da minha voz”) reflete algo subjetivo, visto que depende do perfil comunicativo e não somente do gênero. Os três itens referem experiências subjetivas, que não necessariamente são vivenciadas ou observadas por todas as pessoas não binárias, o que pode ter levado ao preenchimento da opção não-aplicável. Apesar disso, considerou-se que a versão adaptada para a cultura brasileira transmite a ideia geral do conteúdo do instrumento.

Dessa forma, todos foram considerados compreensíveis para a população de pessoas não binárias e traduziram possíveis experiências relacionadas à voz. Não foi necessária a realização de novos ajustes após a aplicação da versão transculturalmente adaptada na população-alvo. A adaptação transcultural realizada neste estudo foi bem sucedida e tornou o VENI aplicável e de fácil compreensão em PB. Trata-se do início do aprimoramento dos instrumentos voltados aos serviços clínicos e de pesquisa com foco na voz e comunicação das pessoas não binárias do Brasil. O instrumento deverá ser submetido à validação para confirmação da estrutura, da validade e da confiabilidade em PB.

## CONCLUSÃO

O VENI foi adaptado transculturalmente para o português brasileiro, resultando na versão denominada Experiências relacionadas a Voz de Pessoas Não Binárias - VENI-Br. Pelas respostas obtidas no pré-teste, considera-se a adaptação transcultural do VENI bem-sucedida.

## Apêndice A Versão final, transculturalmente adaptada do *Voice-related Experiences of Nonbinary Individuals – VENI*

Experiências relacionadas a Voz de Pessoas Não Binárias - VENI-Br

Chave de resposta:

1 = nunca ou quase nunca

2 = as vezes

3 = frequentemente

4 = quase sempre ou sempre

**Table:** Para cada uma das afirmações a seguir, marque a resposta que melhor representa a sua experiência como uma pessoa não binária

1. A qualidade da minha voz varia durante o dia.	**1**	**2**	**3**	**4**
2. É difícil controlar o tom (fino ou grosso) da minha voz.	**1**	**2**	**3**	**4**
3. Algumas emoções mudam o tom (fino ou grosso) da minha voz sem o meu controle.	**1**	**2**	**3**	**4**
4. Minha voz muda do nada em algumas situações.	**1**	**2**	**3**	**4**
5. O tom (fino ou grosso) da minha voz fica pior ao final do dia.	**1**	**2**	**3**	**4**
6. Eu sinto tensão para fazer com que minha voz fique do jeito que eu quero.	**1**	**2**	**3**	**4**
7. Eu faço esforço e preciso prestar atenção para a minha voz ficar como eu quero.	**1**	**2**	**3**	**4**
8. Eu falo em público menos do que eu gostaria por causa da minha voz.	**1**	**2**	**3**	**4**
9. Eu acho que não me identificam como uma pessoa não binária por causa da minha voz.	**1**	**2**	**3**	**4**
10. Eu falo menos do que eu gostaria com pessoas próximas por causa da minha voz.	**1**	**2**	**3**	**4**
11. Eu acho que as pessoas reagem negativamente a minha voz.	**1**	**2**	**3**	**4**
12. Minha voz me atrapalha para eu viver como eu sou.	**1**	**2**	**3**	**4**
13. Eu não gosto do som da minha voz.	**1**	**2**	**3**	**4**
14. Eu sinto que os outros não me levam tão a sério por causa da minha voz.	**1**	**2**	**3**	**4**
15. Eu sinto que os outros pensam mal de mim por causa da minha voz.	**1**	**2**	**3**	**4**
16. Eu me sinto desconfortável ao telefone porque podem me confundir.	**1**	**2**	**3**	**4**
17. Eu me preocupo como as pessoas estranhas percebem a minha voz.	**1**	**2**	**3**	**4**

Itens Físicos: 1, 2, 3, 4, 5, 6, 7

Itens Funcionais: 8, 9, 10, 11, 12, 14, 15, 16

Itens Emocionais: 13, 17
